# Health-Related Quality of Life in Predominantly Young Parental Living Liver Donors: A Cross-Sectional Study in China

**DOI:** 10.3389/fmed.2021.726103

**Published:** 2021-10-01

**Authors:** Yue-Xian Shi, Hai-Ming Zhang, Jing Chen, Ya-Qi Huang, Ming-Ming Yu, Yin-Hui Jin, Wen-Ru Wang, Wei Gao

**Affiliations:** ^1^School of Nursing, Peking University, Beijing, China; ^2^Liver Transplantation Center, Clinical Center for Pediatric Liver Transplantation, National Clinical Research Center for Digestive Diseases, Beijing Friendship Hospital, Capital Medical University, Beijing, China; ^3^Department of Liver Transplantation, Tianjin First Center Hospital, Tianjin, China; ^4^School of Nursing, Tianjin Medical University, Tianjin, China; ^5^Center for Evidence-Based and Translational Medicine, Zhongnan Hospital of Wuhan University, Wuhan, China; ^6^Alice Lee Centre for Nursing Studies, Yong Loo Lin School of Medicine, National University of Singapore, Singapore, Singapore

**Keywords:** quality of life, living donors, liver transplantation, physical function, psychologic status

## Abstract

**Objective:** The health-related quality of life (HRQoL) of donors deserves attention and must be considered for a long time. Many of the published studies had small sample sizes, and research from mainland China, in particular, is scant. Thus, this study aimed to investigate the HRQoL of living liver donors and identify the influencing factors of the HRQoL in mainland China.

**Methods:** This is a cross-sectional study. The data were collected from the liver transplantation center, the Tianjin First Center Hospital, China. Living liver donors older than 18 years and at a minimum of 1-month, post-donation was included. The HRQoL was evaluated using the Medical Outcome Study Short form 36 (SF-36). Sociodemographic and clinical-related variables, HRQoL status, and its potential impact factors were analyzed.

**Results:** A total of 382 living liver donors completed the survey. The median number of months post-donation was 25, and parental donors (99.2%) were the most frequent relationship. The majority of the participants (372, 97.4%) donated their left lateral lobes. Thirty-two (8.4%) donors suffered complications, and of them, 7 suffered from biliary leakage (1.8%), which was the most common one in this study. The physical functioning (PF), role–physical (RP), bodily pain (BP), general health (GH), social functioning (SF), role–emotional (RE), and mental health (MH) scores among the living liver donors were significantly better than those of the Chinese norms. Short-time post-donation [odds ratio (*OR*): 0.008; *p* < 0.001] and male recipients (*OR*:0.195; *p* = 0.024) were associated with the likelihood of a poor physical related quality of life.

**Conclusions:** Despite, in general, good HRQoL outcomes, we also believed that liver donation has an obvious influence on the physical functions of liver donors. More attention and long-term follow-ups are necessary for donors at higher risk based on identified influencing factors and correlates.

## Introduction

The number of living donor liver transplantations has increased annually worldwide. Global Observatory Donation Transplantation (GODT) data (http://www.transplant-observatory.org/) reported that 35,784 liver transplantations had been completed worldwide in 2019, with 21.3% of them being from living liver donors. In China, 5,842 liver transplantations were performed in 2020 and nearly 15% were living liver donor transplantations (http://www.transplant-observatory.org/). A living liver donation has become an important source of liver transplantation to fill the discrepancy between the need and availability of organs.

In a living liver donation, a healthy person donates a fragment of their liver to a liver recipient. Therefore, the safety and well-being of living liver donors must be a first priority.

A wide range of complication rates have been reported in donors after living donor liver transplantations, reaching up to 78.3% in the right lobe and 18% in the left lobe living donor procedures (1, 2). Furthermore, 1.9–14.3% of living liver donors experienced biliary problems, with bile leak being presented approximately 5% of these donors (3, 4). Additional complications observed in liver donors included intra-abdominal hemorrhage and abdominal incision infection. These reported complications can affect the health-related quality of life (HRQoL) of donors to varying degrees. Therefore, the HRQoL of living liver donors has become a research focus.

Previous studies have focused on the occurrence or recovery time, or both of impaired physical function and poor psychological status of living liver donors post-donation. The Adult-to-Adult Living Donor Liver Transportation Cohort Study (A2ALL) from the United States of America (USA) reported that living liver donors developed psychological dysfunction as late as 5 years after donation (5). A study from India assessed the quality of life of 200 living liver donors using the Medical Outcome Study Short form 36 (SF-36), which showed that the physical function scores of donors at 1 year after surgery were worse than their mental state scores (6). Our previous systematic review examined 13 prospective longitudinal studies on the quality of life of living liver donors before and after donation. The results of this study indicated that decreased physical function was sustained for longer than 2 years post-donation, while impaired the social and psychological-related quality of life affected donors for 1–3 months after their liver donations (7).

Some studies compared the HRQoL of living liver donors with the general population by reporting the score of each subscale of the Medical Outcomes Study Short Form-36 (SF-36). Studies from Iran (8), Japan (9), Canada (10), and Germany (11) indicated that the overall HRQoL of living liver donors was significantly better than that of the matched sample from the general population of their own countries. However, when further analyses of each subscale of SF-36 were performed, different findings were revealed. Benzing et al. (11) found a significantly higher score only in the general health perception domain of SF-36. Dew et al. (12) found that the mentally related quality of life score was poorer than the normative mean. Shen et al. (13) found that living liver donors at 1 and 2 years after donation had poorer HRQoL in the physical functioning (PF), role–physical (RP), vitality (VT), and mental health (MH) domains compared with those of the general population of Taiwan.

Investigating the predictors of HRQoL is also important for identifying high-risk groups and promoting the overall welfare of donors. Based on the literature review, the predictors of the HRQoL in living liver donors were summarized. Older age (14), female sex (12, 14, 15), education level of less than a Bachelor degree (16, 17), experiencing one or more complications (18), longer post-donation hospitalization (12, 15, 19), and recipient death (6, 15–17) were predictors of both poor physically and mentally related quality of life scores. Older age (6), Hispanic ethnicity (16), longer time since donation (16), higher body mass index (BMI) (6, 12), experiencing problems with health or life insurance (12), and a family discouraging donation (15) was associated with a higher likelihood of a poor physical score. The education level of a graduate degree (10) and burdensome financial costs (12) also significantly increased the likelihood of a poor mental score.

From the literature review, inconsistent conclusions were found regarding the status of HRQoL. Many of these studies had small sample sizes, and research from mainland China, in particular, was scarce. Therefore, the purpose of the present study was to [1] investigate the HRQoL of living liver donors and [2] analyze the factors that influence the HRQoL of living liver donors in mainland China. Based on the results of previously published studies, it was hypothesized the following:

—Every aspect of the HRQoL of living liver donors is equal to or better than that of the general population.
—The HRQoL of living liver donors is influenced by age, gender, education level, time since donation, BMI, recipient prognosis, types of donated graft, and complications.

## Methods

### Study Design

This was a cross-sectional study.

### Participants

Data were collected from August 2017 to February 2019 in the Tianjin First Center Hospital, a liver transplantation center in China. The study sample comprised all living liver donors treated at this center. The inclusion criteria were, namely, living liver donors older than 18 years old, can understand Chinese, and were at a minimum of 1-month post-donation. Donors with limited abilities of self-expression or those with any physical or mental condition that made them unable to complete the questionnaires were excluded. Human subject approval was obtained from the Peking University Institutional Review Board (No. IRB00001052-19005). All participants signed informed consent forms after receiving a detailed explanation of the purpose and nature of the study.

### Measures

(1) Sociodemographic information. A list derived from a self-reported donor survey developed by the researchers was used to collect demographic variables (age, gender, ethnicity, marital status, education experience, medical payment form, and per capita income of the household).
(2) All donor clinical-related variables, such as months post-donation, relationship to the recipient, type of graft, perioperative complications, and recipient prognosis, were obtained from the electronic medical record system. All complications were graded according to the Clavien–Dindo classification system (20). The BMI was calculated using the recent self-reported height and weight data of the donors. The classification of BMI adopted from the Chinese obesity working group (21) was used. Furthermore, a BMI < 18.5 kg/m^2^ was defined as underweight, a BMI of 18.5–23.9 kg/m^2^ was defined as normal, a BMI of 24–27.9 kg/m^2^ was defined as overweight, and a BMI of ≥28 kg/m^2^ was defined as obese.
(3) The HRQoL of living liver donors was evaluated using the SF-36 questionnaire (version 1), which contains eight subscales, namely, PF, RP, bodily pain (BP), general health (GH), VT, social functioning (SF), role–emotional (RE), and mental health (MH). These subscales are summarized by two-component summary scores, namely, the physical component summary (PCS) and the mental component summary (MCS) scores. The scores of the eight subscales were compared with the Chinese norms (22). Standardized 0–100 scores were converted after calculating the raw scores of each subscale. The PCS and MCS scores were calculated according to the general formula (23). A Mandarin Chinese version of SF-36 was used, which was confirmed as valid and reliable in the Chinese general population. Internal consistency was acceptable for all subscales (Cronbach's α coefficients of 0.51–0.92) (24).

### Data Collection

The investigations were conducted by the first three authors and the doctors or nurses who worked at the follow-up center of the organ donation department that the donor was being treated at. Two methods were used to collect the data. The first was the Wen Juan Xing (https://www.wjx.cn/, Wenjuanxing Tech Co. Ltd., Changsha, China), a professional online questionnaire tool. After each subscale was input into Wen Juan Xing, a quick response code of the online questionnaire was generated. The participants then scanned the code using a cell phone application, WeChat (an instant messaging application), and completed the scale. After the participants completed all the questions, they clicked the “submit” button. The second method comprised a paper scale provided to the participants, which was mainly used for in-hospital donors. The questionnaire was sent to the donors on the spot, and the donors returned it immediately after finishing the questionnaire.

### Statistical Analysis

The Statistical Package for the Social Sciences version 24.0 (IBM Corp., Armonk, NY, USA) was employed to enter and analyze the data. Continuous data were described using the mean and SD for normally distributed variables and otherwise by the median and interquartile range. Categorical variables were described using frequencies and percentages. Different sociodemographic- or clinical-related variable groups were compared using Student's *t*-test and a one-way ANOVA. A *post-hoc* least significant difference (LSD) *t*-test was adopted to perform multiple comparisons. A Spearman's rank correlation analysis was adopted to explore the correlation between the two continuous variables. The factors influencing the PCS and MCS scores were analyzed using multiple linear regressions. Sociodemographic- or clinical-related variables were treated as independent variables. A *p* < 0.05 was considered statistically significant.

## Results

**Figure 1:**
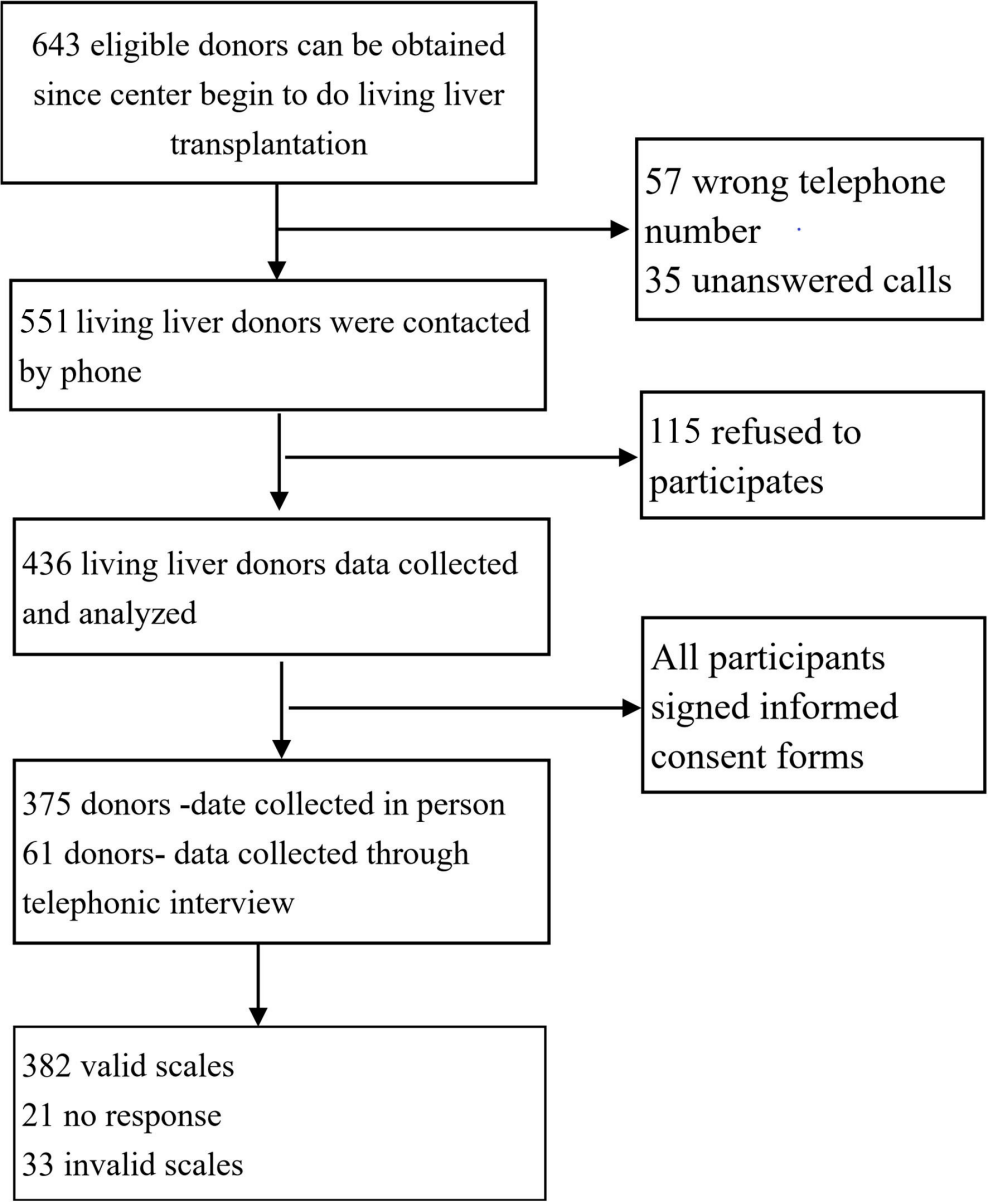
Flow diagram for participants recruitment.

Figure 1 shows the recruitment procedure. Of the 643 donors who donated between 2011 and 2018, 92 donors had incorrect telephone numbers or the phone was unanswered and 115 donors refused to participate in the investigation. A total of 436 donors agreed to participate, subsequently signing informed consent forms after receiving a detailed explanation. Of all the questionnaires distributed, 415 were received and 382 were completely filled out. Therefore, the response rate was 95.18% and the effective rate was 92.05%. Among the 261 donors who had invalid phone numbers, refused to participate in the study, or were offered incomplete questionnaires, 122 (46.7%) were male donors and 139 (53.3%) were female donors. A chi-squared test showed that there was no significant difference between the non-participants and participants based on gender (χ^2^ = *0.3*92, *p* = 0.53). The median age of the non-participants was 32 years old (range: 28–36) and no significant difference was found between the non-participants and participants based on age.

### Subject Characteristics

Tables 1, 2 display the demographic and clinical characteristics of all the participants. The median age of the donors was 32 years, among which 55.8% were female. In addition, most of the donors were of Chinese Han ethnicity (91.6%) and were married (94%). The majority of recipients (93.7%) of our donors at the time of operation were equal or less than 24 months and 44.8% of them were male. The median number of months post-donation was 25 and nearly half of the donors (47.6%) completed their donations 1–3 years previously. Parent donation (99.2%) was the most frequent relationship. With regard to the type of graft, 372 (97.4%) liver donors donated a left lateral lobe, 9 (2.3%) donors donated a left lobe, and only 1 (0.5%) case donated a right lobe graft without the middle hepatic vein.

**Table 1:** Demographic characteristics of living liver donors.

**Variables**		**Total (*N* = 382)**
Age of donor, years (median [IQR])		32.00 (28.25, 36.00)
**Age group of donor (%)**		
	<30 years	125 (32.7)
	30~39 years	217 (56.8)
	40~49 years	35 (9.2)
	≥50 years	24(6.3)
**Gender of donor (%)**		
	Male	169 (44.2)
	Female	213 (55.8)
**Age group of recipients (%)**		
	≤ 6 months	70 (18.3)
	7~12 months	249 (65.2)
	13~18 months	34 (8.9)
	19~24 months	5(1.3)
	>2~15 years	24(6.3)
**Gender of recipients (%)**		
	Male	171 (44.8)
	Female	211 (55.2)
**Race (%)**		
	Han nationality	350 (91.6)
	Other	32 (8.4)
**Marital status (%)**		
	Married	359 (94.0)
	Divorced	18 (4.7)
	Remarriage	5 (1.3)
**Education (%)**		
	Primary school	8 (2.1)
	Junior high	117 (30.6)
	Senior high	107 (28.0)
	College and above	150 (39.3)
**Work Situation**, ***n*** **(%)**		
	Full time job	130 (34.0)
	Temporary work	57 (14.9)
	Self-employed	67 (17.5)
	Farmer	67 (17.5)
	Unemployed	61 (16.0)
**Payment form of medical expenses (%)**		
	Self-paying	131 (40.8)
	Medical insurance for urban workers/	
	urban residents	110 (13.6)
	Cooperative medical insurance	141 (36.9)
**Per capita household income (%)**		
	< ¥2,000Yuan/month	89 (23.3)
	¥2,000~3,999 Yuan/month	174 (45.5)
	¥4,000 Yuan/month	119 (31.2)

*IQR, interquartile ranges*.

**Table 2:** Clinical characteristics of living liver donors.

**Variables**		**Total** **(*N* = 382)**
Months post donation (median [IQR])		25.00 (13.00, 48.00)
**Time since donation surgery (months)**		
	≤ 12	80 (20.9)
	>12–24	93 (24.3)
	>24–36	89 (23.3)
	>36–48	57 (14.9)
	>48–60	37 (9.7)
	>60	26 (6.8)
**Relation to recipients, donor is:** ***n*** **(%)**		
	Parent	379 (99.2)
	Grandparent	3 (0.8)
**Types of graft**, ***n*** **(%)**		
	left lateral lobe	372 (97.4)
	Left lobe	9 (2.3)
	Right lobe graft without middle hepatic vein	1 (0.3)
BMI (median [IQR])		22.42 (20.03, 24.43)
BMI, *n* (%)	<18.5	36 (9.4)
	18.5–23.9	230 (60.2)
	24–27.9	96 (25.1)
	≥28	20 (5.2)
**Recipient prognosis**, ***n*** **(%)**		
	Survived	378 (99.0)
	Died	4 (1.0)
Perioperative complications, *n* (%)	No	347 (91.6)
	Yes	32 (8.4)
Bile leakage		7 (1.8)
Poor/delayed incision healing		6 (1.6)
Seroperitoneum		4 (1.0)
Incision infection		4 (1.0)
Liver cross section effusion		3 (0.8)
Pancreatitis		3 (0.8)
Intestinal obstruction		2 (0.5)
Mild biliary duct dilatation		1 (0.3)
Abdominal infection		1 (0.3)
Gastric retention		1 (0.3)
Clavien grading of morbidity		
	Grade I	7 (1.8)
	Grade II	20 (5.2)
	Grade IIIa	5 (1.3)
SF-36 (mean ± SD)	Physical component summary	49.99 ± 0.85
	Mental component summary	50.00 ± 0.95

*SD, standard deviation; IQR, interquartile ranges; BMI, body mass index*.

According to the Clavien-Dindo classification system, 6 donors experienced poor or delayed incision healing and a donor experienced gastric retention, which was classified as Clavien Grade I. Twenty donors (5 with incision or abdominal infection, 3 with pancreatitis, 2 with intestinal obstruction, 1 with mild biliary duct dilatation, 4 with bile leakage, 2 with seroperitoneum, and 3 with liver cross-section effusion) were classified as Clavien Grade II. Three donors with bile leakage and 2 donors with seroperitoneum were treated using endoscopic retrograde cholangiopancreatography (ECRP) or percutaneous drainage and were thus classified as Clavien Grade IIIa. No donors were classified as Clavien Grade IIIb or had more severe complications. In addition, all complications were cured. Four recipients (1%) died in our investigation (Table 2).

### Generic HRQoL of Living Liver Donors

Table 3 shows the scores of the eight subscales of SF-36. The PF, RP, BP, GH, SF, RE, and MH scores among the living liver donors were significantly better than those of the Chinese norms (Table 3). The mean PCS and MCS scores were 49.99 ± 0.85 and 50 ± 0.95, respectively. The results from the univariate analysis showed that female donors had lower MH scores than male donors (*t* = 2.326, *p* = 0.021). Donors to female recipients had significantly higher scores for PF (*t* = −2.752, *p* = 0.006), RP (*t* = −2.054, *p* = 0.041), and PCS (*t* = −2.687, *p* = 0.008) than donors to male recipients. The results of the one-way ANOVA showed that donors with higher monthly per capita household incomes had significantly high scores for PF (*F* = 3.275, *p* = 0.039) and RP (*F* = 4.307, *p* = 0.014). Specifically, the donors with monthly per capita household incomes ≥4,000 ¥ had higher PF scores than those with monthly per capita household incomes <2,000 ¥ and 2,000–3,999 ¥. The donors with monthly per capita household incomes of 2,000–3,999 ¥ or ≥4,000 ¥ had higher RP scores than those with per capita household incomes <2,000 ¥ (Table 4). Only one donor donated a right lobe graft without the middle hepatic vein; therefore, this case was not included in the univariate analysis. The quality of life of the donors who donated left lobes and left lateral lobes were compared. The results showed that the donors who donated left lobes had lower RP (*t* = 2.031, *p* = 0.043) and PCS (*t* = 2.348, *p* = 0.019) scores than donors who donated left lateral lobes. The donors with preoperative complications had lower scores for BP (*t* = 2.011, *p* = 0.045) than donors without complications (Table 5).

**Table 3:** Comparing the health-related quality of life (HRQoL) with the general Chinese norms.

**SF-36 subscales**	**Present study** **(*N* = 382)**	**The chinese norm**	***t***	***P***
Physical functioning	93.08 ± 8.52	87.92 ± 16.98	11.827	<0.001
Role-physical	84.16 ± 31.02	77.50 ± 34.86	4.197	<0.001
Bodily pain	87.97 ± 15.78	82.22 ± 16.98	7.118	<0.001
General health	77.38 ± 20.27	62.51 ± 17.88	14.338	<0.001
Vitality	69.88 ± 18.23	68.17 ± 17.63	1.836	0.067
Social functioning	89.67 ± 16.94	80.67 ± 19.98	10.392	<0.001
Role emotional	80.45 ± 32.07	67.86 ± 39.44	7.675	<0.001
Mental health	70.94 ± 18.30	68.47 ± 16.90	2.641	0.009

*SF-36, Medical Outcome Study Short form 36*.

**Table 4:** The score of each subscale and two summary domains in SF-S6 for different demographic groups.

**Variables**	**SF-36 (8 Subscales)**								**SF-36 (2 Domains)**	
	**PF**	**RP**	**BP**	**GH**	**VT**	**SF**	**RE**	**MH**	**PCS**	**MCS**
**Gender of donor**										
Male	93.49 ± 7.88	83.88 ± 30.90	88.17 ± 14.89	78.54 ± 21.03	70.83 ± 17.76	88.23 ± 19.00	81.46 ± 31.88	73.37 ± 17.54	50.00 ± 0.84	50.06 ± 0.89
Female	92.75 ± 9.00	84.39 ± 31.19	87.80 ± 16.48	76.46 ± 19.65	69.13 ± 18.60	90.82 ± 15.04	79.66 ± 32.27	69.01 ± 18.69	50.00 ± 0.87	49.96 ± 0.99
*t*	0.848	−0.161	0.227	0.993	0.903	−1.486	0.546	**2.326**	0.054	1.015
*P*	0.397	0.872	0.821	0.321	0.367	0.138	0.586	**0.021**	0.957	0.311
**Gender of recipients**										
Male	91.75 ± 9.32	80.56 ± 35.33	86.54 ± 16.65	75.89 ± 21.49	68.60 ± 18.10	90.38 ± 15.24	77.97 ± 34.52	69.96 ± 17.95	49.87 ± 0.98	50.00 ± 0.90
Female	94.15 ± 7.67	87.09 ± 26.76	89.12 ± 14.97	78.59 ± 19.19	70.92 ± 18.31	89.10 ± 18.21	82.46 ± 29.87	71.73 ± 18.57	50.10 ± 0.72	50.00 ± 0.99
*t*	**−2.752**	**−2.054**	−1.589	−1.297	−1.242	0.736	−1.363	−0.940	**−2.687**	−0.093
*P*	**0.006**	**0.041**	0.113	0.195	0.215	0.462	0.174	0.348	**0.008**	0.926
**Race**										
Han	93.19 ± 8.53	84.21 ± 30.91	87.77 ± 15.79	77.52 ± 20.33	70.24 ± 18.06	89.56 ± 16.90	80.67 ± 31.98	70.97 ± 18.44	50.00 ± 0.86	50.00 ± 0.95
Other	94.67 ± 5.81	88.33 ± 28.14	92.67 ± 11.75	80.60 ± 18.06	64.67 ± 19.59	95.56 ± 8.19	77.78 ± 32.53	70.93 ± 11.85	50.27 ± 0.43	49.90 ± 0.73
*t*	2.713	0.302	1.366	1.576	**3.046**	1.930	0.594	2.280	1.869	1.897
*P*	0.045	0.824	0.253	0.195	**0.029**	0.124	0.619	0.079	0.134	0.130
**Marital status**										
Married	93.02 ± 8.48	84.19 ± 31.14	87.91 ± 15.86	77.58 ± 20.24	70.28 ± 17.96	89.38 ± 17.09	80.50 ± 32.06	71.29 ± 18.09	50.00 ± 0.84	50.01 ± 0.94
Divorced	93.06 ± 10.17	81.94 ± 32.99	89.78 ± 16.09	71.28 ± 22.12	61.67 ± 22.23	92.59 ± 15.24	74.07 ± 35.34	64.22 ± 22.14	50.04 ± 1.16	49.68 ± 1.12
Remarriage	97.00 ± 4.47	90.00 ± 13.69	85.20 ± 9.34	85.00 ± 12.55	71.00 ± 19.49	100.00 ± 0.00	100.00 ± 0.00	70.40 ± 16.88	50.19 ± 0.31	50.31 ± 0.76
*F*	0.536	0.134	0.197	1.188	1.931	1.251	1.287	1.282	0.143	1.332
*P*	0.585	0.875	0.822	0.306	0.146	0.287	0.277	0.279	0.867	0.265
**Education**										
Elementary school	94.38 ± 6.23	65.62 ± 42.13	89.00 ± 12.19	73.62 ± 25.99	65.00 ± 19.09	87.50 ± 18.25	79.17 ± 39.59	69.50 ± 7.98	49.85 ± 0.71	49.88 ± 0.45
Junior high	92.82 ± 8.37	83.12 ± 32.80	88.03 ± 16.51	77.98 ± 21.01	68.76 ± 19.96	90.69 ± 17.32	80.06 ± 33.64	72.62 ± 18.59	49.96 ± 0.87	50.05 ± 0.93
Senior high	92.43 ± 9.60	82.01 ± 31.62	88.34 ± 15.38	78.10 ± 19.71	68.60 ± 17.43	89.51 ± 13.81	78.19 ± 34.60	70.32 ± 18.64	49.98 ± 0.90	49.96 ± 0.99
College and above	93.67 ± 7.94	87.50 ± 28.19	87.59 ± 15.77	76.60 ± 19.93	71.93 ± 17.29	89.11 ± 18.63	82.44 ± 28.56	70.16 ± 18.26	50.05 ± 0.81	50.00 ± 0.96
*F*	0.540	1.757	0.059	0.244	1.150	0.242	0.378	0.474	0.352	0.226
*P*	0.655	0.155	0.981	0.866	0.329	0.867	0.769	0.701	0.788	0.878
**Work situation**, ***n*** **(%)**										
Full time job	93.42 ± 8.56	87.88 ± 28.40	88.28 ± 15.48	77.03 ± 18.18	71.23 ± 17.50	89.23 ± 17.77	82.82 ± 28.53	70.03 ± 19.03	50.06 ± 0.82	49.99 ± 0.97
Temporary work	92.98 ± 8.28	86.84 ± 28.39	89.28 ± 13.40	79.49 ± 19.80	70.09 ± 20.71	89.47 ± 18.83	85.96 ± 30.18	73.33 ± 17.49	50.02 ± 0.79	50.12 ± 0.94
Self-employed	94.55 ± 6.32	84.70 ± 30.76	86.31 ± 17.28	75.55 ± 22.37	68.66 ± 16.91	88.39 ± 16.57	75.62 ± 36.97	70.21 ± 16.93	50.06 ± 0.75	49.85 ± 0.90
Farmer	90.90 ± 10.07	79.10 ± 33.60	88.24 ± 13.68	79.66 ± 21.73	69.78 ± 18.02	90.88 ± 17.61	78.61 ± 30.54	72.36 ± 18.11	49.86 ± 0.94	50.11 ± 0.94
Unemployed	93.20 ± 8.71	78.69 ± 35.31	87.59 ± 18.93	75.67 ± 21.13	68.28 ± 19.28	90.89 ± 12.75	77.60 ± 36.37	69.90 ± 19.34	49.94 ± 0.99	49.95 ± 0.98
*F*	1.670	1.507	0.307	0.617	0.371	0.282	1.157	0.498	0.764	0.899
*P*	0.156	0.199	0.873	0.650	0.829	0.890	0.330	0.737	0.549	0.465
**Payment form of medical expenses**										
Self-paying	91.96 ± 9.29	82.60 ± 32.82	87.48 ± 14.66	76.09 ± 21.73	68.38 ± 18.64	87.01 ± 20.38	78.60 ± 32.53	68.49 ± 20.51	49.94 ± 0.86	49.89 ± 1.04
Cooperative medical insurance	93.22 ± 8.67	82.01 ± 32.89	87.84 ± 16.05	78.79 ± 19.97	69.47 ± 18.71	90.91 ± 14.19	80.30 ± 32.42	73.40 ± 17.38	50.13 ± 0.89	50.06 ± 0.81
Medical insurance for urban workers/urban residents	94.51 ± 6.84	89.22 ± 25.00	88.83 ± 17.06	77.44 ± 18.46	72.60 ± 16.79	91.94 ± 14.09^**$**^	83.33 ± 31.03	71.33 ± 15.55	49.97 ± 0.79	50.08 ± 0.93
*F*	2.759	1.867	0.228	0.618	1.675	**3.126**	0.658	2.563	1.604	1.695
*P*	0.065	0.156	0.797	0.539	0.189	**0.045**	0.519	0.078	0.203	0.185
Per capita income of household										
< ¥2000Yuan/month	91.85 ± 9.72	75.84 ± 36.63	86.53 ± 16.09	77.47 ± 23.33	65.96 ± 21.35	89.39 ± 19.67	77.53 ± 35.81	70.34 ± 20.67	49.84 ± 0.97	49.96 ± 1.11
¥2000~3999 Yuan/month	92.61 ± 8.51	87.21 ± 28.79^▴^	88.70 ± 14.49	77.91 ± 18.37	70.86 ± 17.27	90.81 ± 13.95	82.38 ± 29.70	71.77 ± 16.96	50.01 ± 0.84	50.07 ± 0.84
≥¥4000 Yuan /month	94.66 ± 7.33^▴^^Δ^	85.92 ± 28.68^▴^	87.97 ± 17.33	76.55 ± 20.61	71.39 ± 16.75	88.24 ± 18.65	79.83 ± 32.54	70.18 ± 18.39	50.10 ± 0.77	49.92 ± 0.96
*F*	**3.275**	**4.307**	0.554	0.160	2.747	0.829	0.704	0.328	2.577	1.030
*P*	**0.039**	**0.014**	0.575	0.852	0.065	0.437	0.495	0.721	0.077	0.358

$ *Compared with payment form of self-paying, p < 0.0.05.*

▴ *Compared with per capita income of household <2,000 yuan/month, p < 0.0.05.*

Δ *Compared with per capita income of household of 2,000–3,999 yuan/month, p < 0.05.*

*SF-36, the Medical Outcomes Study Short Form-36; PF, physical functioning; RP, role–physical; BP, bodily pain; GH, general health.*

*VT, vitality; SF, social functioning; RE, role–emotional; MH, mental health; PCS, physical component summary.*

*MCS, mental component summary.*

*Bold represents statistically significant*.

**Table 5:** The score of each subscale and two summary domains in SF-S6 for different clinical groups.

**Variables**	**SF-36 (8 Subscales)**								**SF-36 (2 Domains)**	
	**PF**	**RP**	**BP**	**GH**	**VT**	**SF**	**RE**	**MH**	**PCS**	**MCS**
**Relation to recipients:** ***n*** **(%)**										
Parent	93.07 ± 8.54	84.23 ± 30.98	87.94 ± 15.80	77.31 ± 20.32	69.91 ± 18.24	89.62 ± 16.99	80.30 ± 32.15	70.85 ± 18.31	49.97 ± 0.86	50.00 ± 0.95
Adult child										
Grandparent	93.33 ± 7.64	75.00 ± 43.30	91.33 ± 15.01	87.00 ± 8.66	66.67 ± 20.21	96.30 ± 6.41	100.00 ± 0.00	82.67 ± 12.86	49.83 ± 0.54	50.64 ± 0.57
*t*	−0.052	0.513	−0.371	−0.825	0.306	0.679	−1.060	−1.115	0.348	−1.175
*P*	0.959	0.608	0.711	0.410	0.760	0.497	0.290	0.266	0.728	0.241
**Types of graft, n (%)**										
Left lateral lobe	93.20 ± 8.50	84.88 ± 30.30	88.22 ± 15.58	77.46 ± 20.02	69.91 ± 18.27	89.96 ± 16.42	80.56 ± 32.02	70.86 ± 18.26	50.02 ± 0.83	49.99 ± 0.94
Left lobe	87.78 ± 8.70	63.89 ± 43.50	80.56 ± 21.06	77.77 ± 29.15	70.56 ± 18.10	78.01 ± 31.64	74.07 ± 36.43	75.56 ± 21.01	49.35 ± 1.28	50.15 ± 1.39
*t*	1.890	**2.031**	1.444	−0.047	−0.105	1.922	0.598	−0.760	**2.348**	−0.463
*P*	0.060	**0.043**	0.149	0.963	0.916	0.055	0.550	0.448	**0.019**	0.643
**Recipient prognosis**										
Survived	93.07 ± 8.54	84.06 ± 31.15	87.92 ± 15.83	77.48 ± 20.21	69.81 ± 18.27	89.83 ± 16.76	80.51 ± 31.92	70.94 ± 18.34	50.00 ± 0.86	50.00 ± 0.95
Dead	93.75 ± 7.50	93.75 ± 12.50	92.00 ± 9.24	68.50 ± 27.44	76.25 ± 14.93	75.00 ± 29.22	75.00 ± 50.00	71.00 ± 15.45	50.16 ± 0.53	49.70 ± 1.29
*t*	−0.159	−0.621	−0.514	0.881	−0.702	1.013	0.342	−0.006	−0.383	0.628
*P*	0.874	0.535	0.608	0.379	0.483	0.385	0.733	0.995	0.702	0.530
**Perioperative complications**										
No	93.07 ± 8.67	84.00 ± 31.12	93.31 ± 12.72	77.40 ± 20.46	70.21 ± 17.99	89.75 ± 16.71	80.29 ± 32.05	70.90 ± 18.16	49.99 ± 0.87	50.01 ± 0.94
Yes	93.13 ± 6.81	85.94 ± 30.41	87.48 ± 15.95	77.16 ± 18.45	66.25 ± 20.64	88.89 ± 19.55	82.29 ± 32.77	71.38 ± 20.07	50.11 ± 0.66	49.94 ± 1.04
*t*	−0.034	−0.338	**2.011**	0.066	1.178	0.274	−0.338	−0.140	−0.732	0.398
*P*	0.973	0.736	**0.045**	0.948	0.240	0.240	0.735	0.889	0.465	0.717

*SF-36, the Medical Outcomes Study Short Form-36; PF, physical functioning; RP, role–physical; BP, bodily pain; GH, general health; VT, vitality; SF, social functioning; RE, role-emotional; MH, mental health; PCS, physical component summary; MCS, mental component summary. Bold represents statistically significant.*

Spearman's rank correlation analysis showed that age correlated positively with PF (*r* = 0.128, *p* = 0.013), GH (*r* = 0.116, *p* = 0.024), VT (*r* = 0.181, *p* < 0.001), and PCS scores (*r* = 0.136, *p* = 0.008), while months post-donation was positively associated with PF (*r* = 0.19, *p* < 0.001), RP (*r* = 0.250, *p* < 0.001), BP (*r* = 0.202, *p* < 0.001), VT (*r* = 0.122, *p* = 0.017), and PCS (*r* = 0.251, *p* < 0.001) scores. The BMI correlated positively with GH (*r* = 0.169, *p* = 0.001), VT (*r* = 0.128, *p* = 0.012), and PCS (*r* = 0.105, *p* = 0.041) scores (Table 6).

**Table 6:** Bivariate correlations for continuous variables (*n* = 382).

**Variable**		**Age of donor**	**Age of recipients**	**Months since donation**	**BMI**
PF	*r*	0.128	−0.028	0.190	0.055
	*P*	**0.013**	0.585	** <0.001**	0.281
PR	*r*	0.067	0.013	0.250	0.075
	*P*	0.191	0.795	** <0.001**	0.141
BP	*r*	0.068	−0.011	0.202	0.071
	*P*	0.186	0.824	** <0.001**	0.164
GH	*r*	0.116	0.017	0.068	0.169
	*P*	**0.024**	0.736	0.187	**0.001**
VT	*r*	0.181	−0.022	0.122	0.128
	*P*	** <0.001**	0.672	**0.017**	**0.012**
SF	*r*	0.036	−0.066	−0.001	0.027
	*P*	0.489	0.195	0.981	0.604
RE	*r*	0.093	0.011	0.076	0.089
	*P*	0.068	0.830	0.137	0.081
MH	*r*	0.077	0.019	0.011	0.080
	*P*	0.132	0.713	0.834	0.119
PCS	*r*	**0.136**	−0.015	0.251	0.105
	*P*	**0.008**	0.770	** <0.001**	**0.041**
MCS	*r*	0.072	−0.019	0.018	0.079
	*P*	0.159	0.716	0.719	0.121

*BMI, body mass index; PF, physical functioning; RP, role–physical; BP, bodily pain; GH, general health; VT, vitality; SF, social functioning; RE, role–emotional; MH, mental health; PCS, physical component summary; MCS, mental component summary.*

*Bold represents statistically significant*.

### Independent Influencing Factors of Physically and Mentally Related Quality of Life

The influencing factors of poor PCS and MCS scores in SF-36 were estimated using multiple linear regressions. However, there were a small number of different complications, therefore, in the multiple regressions, the combined data of complications, i.e., the presence or absence of complications as an independent variable, were used. Meanwhile, there was a very limited number of donors who donated left lobe or right lobe grafts as variables for “type of graft” and few grandparents donors as variables for “relation to recipients”. The worse prognosis of recipients as a variable for “recipient prognosis” was also lacking. Therefore, these two variables were not included in the multivariate analysis. Two factors emerged from the regression analysis: short-time post-donation [odds ratio (*OR*): 0.008; *p* < 0.001] and male recipients (*OR*: 0.195; *p* = 0.025) were associated with the likelihood of a poor PCS (Table 7). No significant influencing factor was found for the MCS score.

**Table 7:** Independent influencing factors of the PCS from multivariate analysis (*N* = 382).

**Dependent variables**	**Independent variables**	***R^**2**^***	**Beta**	**Std**	**Standard beta**	***t***	***P***
PCS		0.285					
	Age of donors		0.004	0.008	0.029	0.549	0.584
	Gender of donors		0.027	0.092	0.016	0.300	0.764
	Age of recipients		−0.001	0.002	−0.021	−0.377	0.706
	**Gender of recipients**		**0.195**	**0.087**	**0.114**	**2.257**	**0.025**
	Race		−0.025	0.156	−0.008	−0.158	0.875
	Work situation		−0.018	0.031	−0.032	−0.588	0.557
	Education		0.014	0.055	0.014	0.245	0.807
	Payment form of medical expenses		0.033	0.055	0.031	0.611	0.542
	Marriage		0.103	0.141	0.037	0.729	0.466
	Per capita household income		0.084	0.063	0.072	1.332	0.184
	Perioperative complications		0.159	0.156	0.052	1.019	0.309
	BMI		0.017	0.014	0.065	1.253	0.211
	**Months post donation**		**0.008**	**0.002**	**0.189**	**3.624**	** <0.001**

*BMI, body mass index; PCS, physical component summary. Bold represents statistically significant*.

## Discussion

This cross-sectional study was conducted in a relatively large sample. With this, we were able to report the HRQoL and its influence factors on living liver donors in China. The first hypothesis was confirmed: the HRQoL of living liver donors was equal to or better than that of the Chinese general population. Specifically, the PF, RP, BP, GH, SF, RE, and MH scores were significantly higher in liver donors than in the Chinese norm, while VT was similar to the Chinese norm. A short-time post-donation and donation to male recipients were independently associated with a poor PCS score, which provided partial support for the second hypothesis.

Yuen et al. (25) reported that the SF-36 domain scores of parental donors were considered average or above average compared with those of the Singaporean population. This was consistent with the results of our study. After experiencing a strict screening program, only an absolutely healthy person could donate their liver and was thus likely to be healthier than the norms. Furthermore, the majority of the participants in this study were young parents of recipients. The greatly improved health statuses of the recipients after receiving liver transplantation could be beneficial for the HRQoL of the donor parents. In a Chinese study, (26) compared the HRQoL of living liver donors with Chinese norms, the results of which demonstrated that the scores for BP and SF were significantly lower in donors compared with those in the general population. In the present study, 70.6% of the donors had undergone donation surgery within 2 years. The short-time post-donation could explain the difference between the results.

Our study indicated that short-time post-donation was independently associated with poor PCS scores. The mean PCS score was 49.99, which was slightly lower than the MCS score. A study from India found that the mean PCS score was 48.76, which was lower than the MCS score among donors at 1-year post-donation (6). The same results were found by Shamsaeefar et al. (8). Since living liver donation surgery is an invasive procedure, decreased physically related quality of life appears to be a common phenomenon in the early postoperative period (13, 27, 28). Our study confirmed this finding. It should be noted that our previous meta-analysis (7) found that, compared with pre-donation, a significant decline in physically related quality of life was sustained up to 2 or more years post-donation, while pain and fatigue existed within half a year after a living liver donation. A study from Ladner (16) reported that a long time since the donation was associated independently with a high score of physical quality of life (16). This suggests that the physical functions of living liver donors require long-term attention. In the short-term post-donation, impaired physical function is manifested as pain and fatigue, while the influence of donation surgery on the physical functions of liver donors, such as activity ability and independence, might exist for a long time.

Interestingly, this study was the first to find that the recipient being male was an independent influencing factor for poor physically related quality of life. This is because, compared with girls, boys have a naughtier nature. As their caregivers, parental donors need to pay more attention to their safety and health status. In terms of taking immunosuppressants and other drugs and reexamining, parental donors might require more supervision and support. However, limited data were collected from the recipients; therefore, the results must be further explored and verified in future studies.

The results of our study showed that female donors had significantly lower MH scores on SF-36 than male donors. This was similar to the results of studies by Morooka et al. (9) and Janik et al. (14), which reported that female donors showed lower MCS scores on SF-36 than male donors. The results of this study, however, were different from those of a previous Chinese study (26), which reported that female donors scored lower than male donors in the GH domain of SF-36. In our study, the majority of the donors were the parents of recipients, and among them, there were more female donors than male donors. After donation surgeries, female donors, in general, bore the heavy responsibility of taking care of their sick children while dealing with the possibility of losing their jobs and social interaction, which, in turn, would affect their mental status.

The relationship between income and the health outcome of organ donation is interactive. Economic status affects the outcome of donation (15), and donation surgery also affects family income (29). Butt et al. (15) found that a higher household income was associated with lower post-donation pain. In our study, univariate analysis showed that donors with monthly per capita household incomes ≤ 2,000 ¥ had lower scores on PF and RP domains of SF-36 compared with donors with high per capita household incomes. Thus, it is necessary to examine more income indicators (e.g., personal income of the donor and the impact of donation surgery on this personal income) to confirm the relationship between income and the HRQoL of liver donors.

Donors donating left lobes were found to have significantly low scores on RP and PCS. To date, few studies have considered the effect of different types of graft on the HRQoL of living liver donors. Takada et al. (18) found no significant differences in physically, mentally, and socially related quality of life scores between right and left lobe donors. Raza et al. (30) indicated that the HRQoL of living liver donors did not differ among different types of donation (left lateral segment, left, or right lobe). In the present study, 97.4% of donors donated left lateral lobes and only one donor donated a right lobe. Thus, the impact of the types of graft on the HRQoL of living liver donors requires confirmation in a future study. However, the relatively large volume of liver lobe donations (such as the right lobe with or without median hepatic vein and the left lobe) in adult-to-adult living donor liver transplantations is almost inevitable. Therefore, the health, safety, and long-term HRQoL of these donors should be prioritized.

In the published literature, post-donation morbidity rates among liver donors vary from 15 to 78.3% (1, 11, 19, 25, 31–38). These complication rates, which vary significantly, were due to the different parts of the liver donated by the included donors. Yi et al. (1) reported that 78.3% of right liver donors experienced postoperative complications, while 14.5% of them had complications of Clavien Grading III or above. Berglund et al. (2) compared the complication rates between right-lobe and left-lobe donors, with the results showing that 48% of the right-lobe donors and 18% of the left-lobe donors (including left lateral segmentectomy and left hepatectomy) had complications. A Chinese study by Sun et al. (34) found that post-operative complications were observed in 40.1% of donors. These included 38.2% of right-lobe donors, 0.6% of left-lobe donors, and 1.3% of left lateral lobe donors. Overall, the morbidity of our study was 8.4%. Of them, 1.3% was accounted for by Clavien Grade IIIa and biliary leakage in our participants was the most common complication (1.8%). The donors in this study were mainly young parents who donated relatively small parts of their livers to their children. Therefore, the damage to these donors was smaller than in adult-to-adult liver transplantations. This study also included many left lateral lobe donors, a type of donation that has been confirmed by studies to have a minor impact on liver donors (2, 36). Surgical complications, even mild ones, can affect physical function, body image, or daily activity to some extent. Complications and their influences on physical function might increase the risk of psychological problems among living liver donors. Similarly, physical problems might be aggravated by psychological problems, resulting in a vicious cycle. A prospective study from Taiwan, China (17), indicated that post-donation complications were a significant predictor of the PCS score, while a study of over 500 donors conducted by Takada et al. (18) reported that the incidence of two or more comorbidities was associated significantly with decreased PCS and MCS scores. This might have been caused by the small sample of complications. Furthermore, the regression analysis in this study did not find any factors affecting the HRQoL of living liver donors.

The association between age and HRQoL is still disputed in published studies. Chandran et al. (6) reported that age above 50 years in living liver donors affected the physically related quality of life scores negatively. Morooka et al. (9) compared physical and mental domain scores in different age groups and reported that the MCS scores of donors aged ≥70 or 60–69 years were better than those of younger subjects. In addition, multiple regression analysis indicated that donor age was associated independently with both the PCS and the MCS scores, in which older age was a negative predictor for the PCS score, but a positive predictor for the MCS score. Janik et al. (14) stated that both the PCS and MCS scores decreased with age. A Chinese study by Jin et al. (26) found that older donors (aged ≥ 40 years old) reported a significantly higher HRQoL in domains such as SF and MH. In our study, a univariate analysis found that age was associated positively with PF, GH, VT, and PCS scores. However, no significant correlations were detected using multiple regression analysis. The majority of the liver donors that participated in our study were parents of pediatric recipients, which meant that many of them were young (89.5% were aged <40 years old). The age distribution was relatively concentrated, which might explain why our research results were different from those of previously published studies.

Published studies have reported that high BMI was associated negatively with the HRQoL of living liver donors. Chandran et al. (6) found that a high BMI had a detrimental effect on the physical quality of life of living liver donors at 1-year post-donation. A prospective, multicenter, and longitudinal study from an A2ALL found that obese donors exhibited significantly poorer physical function scores (12). However, in our study, only a simple correlation analysis found a positive correlation between BMI and the GH, VT and PCS scores, which were inconsistent with previous studies. This might reflect the fact that weight and height were self-reported by the donors. Moreover, BMI is one of the easiest indicators to obtain and thus could be used to estimate the body weight of living liver donors. However, in terms of assessing the effect of obesity on physical function or mental status, BMI is not an ideal indicator of obesity. It may be possible that this correlation can be confirmed by specific and sensitive indicators, such as the waist-hip ratio or triceps skinfold thickness, in future studies.

### Strengths and Limitations

This study has obvious strengths in that the HRQoL and influencing factors of living liver donors were reported using a large sample in mainland China. The study had a high rate of questionnaire response and data validity. Therefore, the sociodemographic data and the donor self-reported data were obtained and analyzed. However, there were several limitations. First, compared with the donors who donated the left lateral segments of their liver, there were too few right lobes and full left lobe liver donors in this study, which would have allowed for a more in-depth comparative analysis to be undertaken. Second, the medical records were retrospectively reviewed to obtain the data for early postoperative complications. However, these data were limited and further affected the results of the PCS and MCS analyses. Third, this was a cross-sectional study with the absence of pre-donation data that performed the collection of single point data only. Therefore, the changes in HRQoL between pre and post-donation or at different time points of post-donation could not be examined. Fourth, the HRQoL of living liver donors was only assessed using SF-36. However, some specific problems and/or minor issues associated with organ transplantation surgery might not be adequately measured using this instrument. The study group is now devoted to developing a quality assessment scale for living organ donors, and they will continue to focus on the HRQoL of living liver donors in research. Finally, the participants were mainly young parental donors, which limited their ability to represent the whole living liver donor population.

## Conclusion

In summary, this study provided information regarding the status and independent influencing factors of the HRQoL in living liver donors. Compared with the Chinese norms from the general population, living liver donors had significantly higher scores on seven subscales of SF-36. Short-time post-donation and donation to male recipients were negatively associated with the poor physical quality of life. Despite the generally good HRQoL outcomes, it is also believed that liver donation has an obvious influence on the physical functions of donors. The study provided valuable information for the management of living liver donors. First, the physical problems of liver donors in the short-term post-donation should be monitored closely and targeted interventions should be given in a timely manner. Continuous follow-ups and surveillance are necessary for long-term post-surgery. The conditions of donors and their recipients should also be assessed regularly. Second, it is suggested that psychological counseling should be considered as a necessary procedure for living liver donors, especially for young female donors. Third, the economic situation of living liver donors is a matter of concern. Social support for donors from low-income families should be strengthened. Last, education relevant to potential donation-related health concerns and negative factors for HRQoL should be provided for liver donors, both pre- and post-donation.

## Data Availability Statement

The original contributions generated for the study are included in the article, further inquiries can be directed to the corresponding author.

## Ethics Statement

The studies involving human participants were reviewed and approved by Peking University Institutional Review Board (No. IRB00001052-19005). The patients/participants provided their written informed consent to participate in this study.

## Author Contributions

Y-XS, H-MZ, and WG contributed to the study design, manuscript preparation, data interpretation, and paper writing. JC, Y-QH, and H-MZ contributed to data collection and statistical analysis. M-MY, Y-HJ, and W-RW contributed to data interpretation, paper revision, and literature retrieval. All authors contributed to the article and approved the submitted version.

## Funding

This work was supported by grants from the National Natural Science Foundation of China (Nos. 71603272 and 71974008), the Fundamental Research Funds for the Central Universities (No. BMU2021YJ025), and the China Postdoctoral Science Foundation (Nos. 2018M641114 and 2020T130029).

## Conflict of Interest

The authors declare that the research was conducted in the absence of any commercial or financial relationships that could be construed as a potential conflict of interest.

## Publisher's Note

All claims expressed in this article are solely those of the authors and do not necessarily represent those of their affiliated organizations, or those of the publisher, the editors and the reviewers. Any product that may be evaluated in this article, or claim that may be made by its manufacturer, is not guaranteed or endorsed by the publisher.
